# Smith’s Minor Surgery

**Published:** 1843-10

**Authors:** 


SMITH'S MINOR SURGERY.

This is a volume of three hundred and three pages 12mo., and is divided into four parts: Part I, treats of the preparation and application of dressings; Part II, of the application of the bandage, simple and compound; Part III, of the apparatus for fractures and dislocations; and Part IV, of minor surgical operations. Under Part II, Dr. Smith introduces the Handkerchief system of Mayor; we do not observe that the volume has any other claim to preference over those already in use, notwithstanding it is endorsed by many of our eastern *confreres.* The part devoted to Minor Surgical Operations is meagre indeed—and might just as well have been omitted. The author acknowledges his draughts upon the treatises of Velpeau, Gerdy, and Mayor; he might have added Cutler, as many of the illustrations of the volume will appear marvellously familiar to one acquainted with Cutler's treatise, issued in similar form by the same publishers.

Touching the subject of Surgical Operations, we would say that we know of no work equal to Tavernier's Operative Surgery, translated by Professor Gross. This is now out of print, at least we have never been able to get a copy of it; and we think it would be doing the profession a service, if some publishing house, Messrs. Barrington and Haswell, for instance, would put forth a second edition. We are confident it would repay them. C.

